# When less is more: shortening the Lpp protein leads to increased vancomycin resistance in *Escherichia coli*

**DOI:** 10.1038/s41429-023-00658-3

**Published:** 2023-09-25

**Authors:** Hannah Wykes, Vuong Van Hung Le, Catrina Olivera, Jasna Rakonjac

**Affiliations:** 1 School of Natural Sciences, Massey University, Palmerston North, New Zealand; 2 Section of Microbiology, Department of Biology, University of Copenhagen, Copenhagen, Denmark

**Keywords:** Antibiotics, Bacterial genetics, Antimicrobial resistance

## Abstract

Vancomycin is a naturally occurring cell-wall-targeting glycopeptide antibiotic. Due to the low potency of this antibiotic against Gram-negative pathogens, such as *Escherichia coli*, there is a limited knowledge about interactions between vancomycin and this group of bacteria. Here, we show that an in-frame 63 bp deletion of the *lpp* gene caused a fourfold increase in vancomycin resistance in *E. coli*. The resulting protein, LppΔ21, is 21 amino acids shorter than the wild-type Lpp, a helical structural lipoprotein that controls the width of the periplasmic space through its length. The mutant remains susceptible to synergistic growth inhibition by combination of furazolidone and vancomycin; with furazolidone decreasing the vancomycin MIC by eightfold. These findings have clinical relevance, given that the vancomycin concentration required to select the *lpp* mutation is reachable during typical vancomycin oral administration for treating *Clostridioides difficile* infections. Combination therapy with furazolidone, however, is likely to prevent emergence and outgrowth of the *lpp*-mutated Gram-negative coliforms, avoiding exacerbation of the patient’s condition during the treatment.

## Introduction

Vancomycin, a naturally occurring glycopeptide antibiotic, exerts an antibacterial effect by binding to the N-terminal d-Ala-d-Ala of the pentapeptide stem of the peptidoglycan precursors and the growing peptidoglycan. This inhibits the peptidoglycan synthesis and causes a synthesis/degradation imbalance, resulting in cellular lysis [1]. Vancomycin is conventionally used as the first line therapy to treat nosocomial infections caused by methicillin-resistant *Staphylococcus aureus* and other Gram-positive β-lactam-resistant bacteria by intravenous infusion [2] or to treat severe *C. difficile* infection by oral administration [3]. Given the clinical importance of vancomycin for treatment of Gram-positive infections, it is not surprising that a vast number of studies have been accumulated regarding the interaction between vancomycin and this bacterial group, whereas this is not the case for Gram-negative bacteria. The outer membrane of Gram-negative bacteria is highly impermeable to large molecules (≥600 Da) [4], which effectively restricts vancomycin (~1449 Da) from reaching its target in the periplasm, thwarting its antibacterial activity. Nonetheless, recent efforts to develop vancomycin-based therapies against Gram-negative pathogens, such as synergistic drug combinations [5] and outer-membrane permeabilising vancomycin analogues [6–8] necessitate investigation into the potential mechanism of vancomycin resistance in Gram-negative bacteria.

To understand the basis of *E. coli* resistance to vancomycin, we isolated mutants having an increased resistance to vancomycin as compared to the parental strain and used whole-genome shotgun sequencing and comparative genome analysis to identify underlying genetic mutations.

## Materials and methods

### Growth conditions

*E. coli* growth media used in this study was Cation-Adjusted Mueller Hinton (CAMH from BD BBL^TM^) in liquid broth or solid agar plates made at 1% agar (Pure Science Ltd.). All strains were grown at 37 °C with shaking at 200 rpm for liquid cultures.

### Isolating the vancomycin-resistant mutants

Vancomycin-resistant mutants of the *E. coli* parental strain BW25113 [9] were selected on CAMH agar containing vancomycin at 1024 μg ml^−1^. Briefly, 100 μl of each of twenty independent overnight cultures was added to 2.5 ml of molten 0.5% CAMH agar (at ~47 °C), vortexed, then poured onto the selective plates. Resistant mutant colonies were observed after 48 h incubation at 37 °C and sub-streaked on non-selective plates (without vancomycin) and incubated overnight at 37 °C. The colonies grown on this non-selective agar were used to make stock cultures for later analyses.

### Antimicrobial susceptibility assays

Vancomycin (GoldBio) minimum inhibitory concentrations (MICs) of *E. coli* strains were determined using broth microdilution and agar dilution methods according to Clinical and Laboratory Standards Institute guidelines [10].

### Growth inhibition checkerboard assays

Growth inhibition checkerboard assays were done to examine the interaction between furazolidone and vancomycin in CAMH broth in a 384-well microplate format as previously described with some modifications [5]. Briefly, twofold serial dilutions of furazolidone and vancomycin were used. Each well contained 5 × 10^5^ cfu ml^−1^, 1% DMSO, and antibiotics in a final volume of 50 μl. The microplates were incubated at 37 °C and the OD_600_ measured after 18 h using a Multiskan^TM^ GO Microplate Spectrophotometer. Each treatment was performed in triplicate.

Fractional inhibitory concentration index (FICI) was calculated using the following equation:

$${{{{{\rm{FICI}}}}}}=\frac{{{{{{{\rm{MIC}}}}}}}_{{{{{{\rm{FZ}}}}}}}\left({{{{{\rm{combination}}}}}}\right)}{{{{{{{\rm{MIC}}}}}}}_{{{{{{\rm{FZ}}}}}}}\left({{{{{\rm{alone}}}}}}\right)}+\frac{{{{{{{\rm{MIC}}}}}}}_{{{{{{\rm{VAN}}}}}}}\left({{{{{\rm{combination}}}}}}\right)}{{{{{{{\rm{MIC}}}}}}}_{{{{{{\rm{VAN}}}}}}}\left({{{{{\rm{alone}}}}}}\right)}$$

Where MIC_FZ_(combination) MIC_VAN_(combination) are the MICs for furazolidone and vancomycin when used in combination and MIC_FZ_(alone) MIC_VAN_(alone) are the MICs for furazolidone and vancomycin when used alone. The lowest FICI values were used to determine interactions: FICI ≤ 0.5 indicates synergy, FICI > 4 indicates antagonism, and 0.5 < FICI ≤ 4 indicates additivity [11].

### Comparative genome analysis

Genomic DNA was extracted from overnight cultures using the DNeasy UltraClean Microbial Kit (Qiagen) and submitted for whole genome sequencing at Massey Genome Service (Massey University, Palmerston North, New Zealand). DNA libraries were prepared using the Illumina DNA Prep kit and sequenced on the Illumina MiSeq™ 2 × 250-base paired-end v2 platform. The sequencing data is available at the National Centre for Biotechnology Information (NCBI) GenBank under the BioProject PRJNA854676. The bioinformatic workflow to map sequencing reads to the BW25113 reference genome (accession number CP009273.1) and call mutations was performed as previously described [12].

### Searching for the LppΔ21 mutation in clinical isolates

The amino-acid sequence encoding the LppΔ21 variant was searched against the NCBI non-redundant protein sequence database using the blastp webserver v2.14.0+ with default parameters [13].

### Modelling the structure of Lpp and LppΔ21 proteins

The structure of the mature Lpp has been solved [14]. The LppΔ21 mutant was modelled in trimeric complexes using ColabFold v1.5.2 set at default parameters [15] and visualised using ChimeraX v1.5 [16].

## Results and discussion

**Fig. 1:**
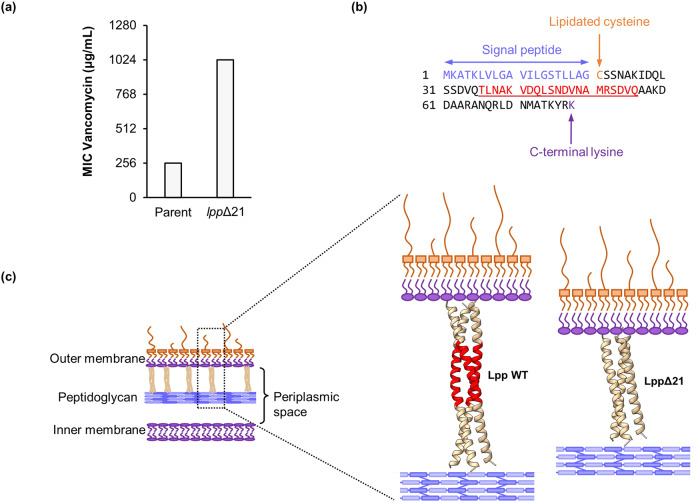
Mutation in *lpp* confers resistance to vancomycin. **a** MIC of the *lpp*Δ21 mutant and parental strain. MIC experiments were conducted using three replicates and yielded identical results. **b** The Lpp protein sequence; the 21 deleted residues in the *lpp*Δ21 mutant are highlighted in red. The N-terminal cysteine (orange) of the mature Lpp protein is lipidated and anchored to the inner leaflet of the outer membrane. The C-terminal lysine (purple) makes a covalent bond to the peptidoglycan. **c** Schematic illustration of a Gram-negative envelope in which the Lpp protein connects the outer membrane to peptidoglycan, dictating the periplasmic space. The trimeric complex of the wild-type Lpp and the LppΔ21 mutant was modelled using ColabFold. The 21 residues deleted from the wild-type Lpp to make the LppΔ21 mutant are coloured in red. WT wild-type

To isolate vancomycin-resistant mutants, 20 independent overnight cultures of *E. coli* (strain BW25113) were each plated on a vancomycin selective plate at 1024 μg ml^−1^. Only one vancomycin-resistant mutant was observed and isolated from the totality of 20 plates. This mutant displayed a vancomycin MIC of 1024 μg ml^−1^, which was fourfold higher than that of the parental strain (256 μg ml^−1^) in liquid broth microdilution assays (Fig. 1a). Whole genome analysis revealed that the mutant had a 63 bp deletion mutation in the *lpp* gene (coordinates 1751783–1751845 in the reference genome BW25113) which led to an in-frame deletion of 21 amino acids in the Lpp protein (Fig. 1b).

Lpp is a highly abundant lipoprotein that folds into a trimeric coiled coil structure (Fig. 1c) [14]. It is present in two forms, transmembrane (free) and periplasmic (bound) [17]. The transmembrane form is exposed on the bacterial surface and has an unknown function, while the periplasmic form anchors its lipidated N-terminus in the periplasm-facing phospholipid leaflet of the outer membrane and covalently links the C-terminal Lys residue to the peptidoglycan (Fig. 1c). This provides the only covalent link between the outer membrane and the peptidoglycan, controlling the width and therefore the overall volume (size) of the periplasmic space [18] and contributing towards the structural integrity and function of the cell envelope [19]. This has been shown through reported properties in *E. coli* expressing an in-frame *lpp* insertion mutant containing an additional 21 amino acids (Lpp^+21^ variant), that was found to have a 3–4 nm longer inter- to outer-membrane distance [18, 20]. Interestingly, a combination of electron cryotomography and molecular dynamics simulations of the cells expressing this mutant showed that the Lpp^+21^ is tilted rather than perpendicular to the membrane surface, minimising the increase in the thickness of the periplasmic space [20]. Furthermore, non-essential genes involved in binding the outer membrane to the peptidoglycan (at a set distance) become essential in this *lpp* mutant, presumably by minimising the increase in thickness of the periplasmic space due to a longer Lpp. These findings demonstrate that increasing the thickness of the periplasm above a limit of about 3–4 nm is lethal to *E. coli*.

In addition, it has been shown that increasing the length of Lpp softened the cell-envelope (decreased stiffness) and rendered *E. coli* more susceptible to vancomycin [18]. By contrast, the Δ*lpp* mutant (i.e. total deletion of the *lpp* coding sequence) and the *lpp*ΔK58 mutant (i.e. deletion of the C-terminal lysine responsible for the peptidoglycan anchorage) were found to have little effect on the vancomycin sensitivity as compared to the wildtype [18]. In our study, we showed a reciprocal effect of the shorter Lpp length on vancomycin susceptibility, where decreasing the Lpp protein length by 21 residues caused a fourfold increase in vancomycin resistance. We speculate that the *lpp*Δ21 mutation may decrease the distance between the outer membrane and the peptidoglycan, decreasing the periplasmic space and thereby increasing the mechanical strength of the cell envelope, resulting in a higher peptidoglycan impairment threshold, and consequently a higher vancomycin MIC required to induce cell death (Fig. 1c). The decreased periplasmic space may also limit the access of vancomycin to its peptidoglycan target by further restricting the already very limited ability of vancomycin to accumulate in the periplasmic space. Alternatively, cells that possess the *lpp*Δ21 variant may have an unusual envelope architecture and, to release envelope stress, produce outer membrane vesicles that can act as a decoy to sequester vancomycin from bacterial cells. Future work looking into the underlying molecular mechanism is warranted.

**Fig. 2:**
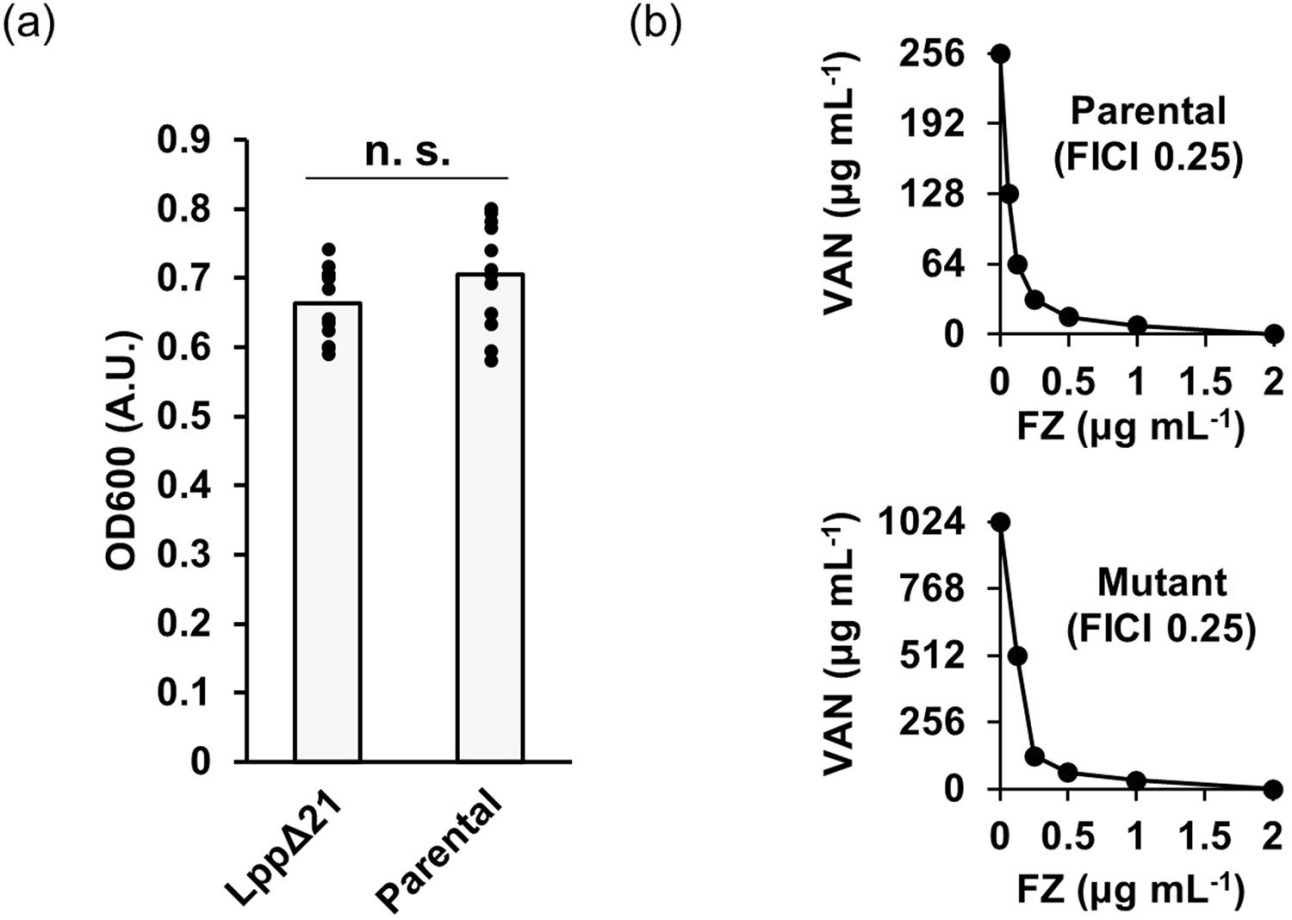
Properties of the *lpp*Δ21 mutant. **a** Bacterial density in overnight cultures is unchanged in the *lpp*Δ21 mutant as compared to the parent strain. The optical density at 600 nm of the culture after 18 h incubation at 37 °C. Twelve replicates of cultures were used. n.s., non-significant, Student’s *t* test (*p* = 0.13, *n* = 12). **b** Synergistic interaction between vancomycin and furazolidone in the *lpp*Δ21 mutant (top graph) and the parent strain (bottom graph). The isobologram curves were generated from the growth inhibition checkerboard assay for the parental strain and vancomycin mutant, performed as described in the “Materials and Methods” section. Each data point indicates the MIC of the drugs, either alone or in combination. Each treatment was performed in triplicate; each replicate yielded identical MICs for individual antibacterials and the combinations

We next asked if the laboratory-selected *lpp*Δ21 mutation is clinically relevant. First, there was no significant difference between the OD_600_ of the mutant and parental strains after 18 h of incubation in CAMH broth at 37 °C (Fig. 2a), indicating that the *lpp* mutation does not significantly impact the *E. coli* growth. In other words, even in the absence of vancomycin selection, the *lpp*Δ21 variant is unlikely to be outcompeted by the parental strain. Second, searching the LppΔ21 amino-acid sequence against the NCBI protein database retrieved one hit belonging to a pathogenic Shiga-toxin producing *E. coli* strain isolated from the USA, designated PNUSAE059255 (Accession number AAVXHG010000001.1). Finding of such a mutant in the database indicates that the vancomycin resistance conferring LppΔ21 variant can be readily selected upon vancomycin exposure. Third, oral vancomycin administration at the dose of 125 or 250–500 mg four times per day is recommended to treat severe or very severe *C. difficile* infections, respectively [3]. With the former dosing, the vancomycin concentrations in the faecal samples, as a proxy for colonic levels, have been reported to reach the range of 50–2000 μg ml^−1^. Furthermore, with a higher dosing regime (500 mg), the faecal vancomycin concentration was consistently higher than 2000 μg ml^−1^ [3]. Opposite to the conventional thought that vancomycin alone does not affect Gram-negative bacteria, it is plausible to argue that the current oral vancomycin treatment for severe *C. difficile* infection may select for vancomycin resistance in gut resident Gram-negative bacteria, including the reported *lpp*Δ21 Shiga-toxin-producing *E. coli* isolate. Vancomycin selection for such a severely pathogenic strain that is shed into the clinical setting and the environment is clearly not desirable in the long term, as it will enrich the environment for this severely pathogenic strain thriving in the gut of vancomycin-treated patients, where nearly all bacteria are wiped out.

The *lpp*Δ21 mutant isolated in this work was further tested for the reported susceptibility to the synergistic effect between vancomycin and furazolidone, a synthetic nitrofuran antibiotic [5]. The synergy was maintained, shown by an unaltered FICI of 0.25 compared to the parental strain (Fig. 2b). This FICI implies vancomycin susceptibility in the *lpp*Δ21 mutant, with the MIC for vancomycin dropping from 1024 to 128 μg ml^−1^ in the presence of 0.25 μg ml^−1^ furazolidone or to 64 μg ml^−1^ in the presence of 0.5 μg ml^−1^ furazolidone (Fig. 2b). This shows that a combined furazolidone-vancomycin treatment regime potentially prevents selection for and/or eradicates the *lpp*Δ21 mutant which is otherwise impossible with vancomycin monotherapy. This finding suggests that a furazolidone-vancomycin combination treatment for *C. difficile* infection could be a potential option to prevent emergence of vancomycin resistance in *E. coli* within the patients’ gut. Since the Lpp homologue can be found in other Gram-negative bacteria and the synergy between furazolidone-vancomycin has been demonstrated previously [5], we speculate that the effect of this combinatorial regime on vancomycin resistance suppression can be generalised to other Gram-negatives, not just limited to *E. coli*, in the gut environment.

In conclusion, we report here a 63-nucleotide in-frame deletion mutation in the *lpp* gene, conferring resistance to vancomycin in *E. coli*. The concentration of vancomycin we used to select the *lpp*Δ21 mutant is reachable during oral vancomycin treatment for *C. difficile* infection. Furthermore, the *lpp*Δ21 mutation was also found in a clinical Shiga-toxin producing *E. coli* isolate, confirming the clinical relevance of the mutation we have isolated. Overall, for those efforts to develop oral vancomycin-based therapies [6–8], we suggest inclusion of the *E. coli lpp*Δ21 mutant to the drug testing panel to evaluate the potential of the developed therapies to escape the known vancomycin resistance in Gram-negative pathogens.
